# Surgical outcomes and quality of life in octogenarians with early-stage non-small cell lung cancer: a prospective cohort study

**DOI:** 10.1016/j.lana.2026.101428

**Published:** 2026-03-13

**Authors:** Louis Gros, Rowena Yip, Wenchao Ma, Jeffrey Zhu, Jiafang Zhang, Sydney Kantor, Siyang Cai, Andrew J. Kaufman, Andrea S. Wolf, Ardeshir Hakami-Kermani, Daniel Nicastri, Dong-Seok Lee, Kimberly J. Song, Brian Housman, David F. Yankelevitz, Emanuela Taioli, Claudia I. Henschke, Raja M. Flores, IELCART Investigators, Raja Flores, Andrew J. Kaufman, Dong-Seok Daniel Lee, Daniel Nicastri, Andrea Wolf, Kimberly Song, Kenneth Rosenzweig, Robert Samstein, Pinaki Dutta, Jorge Gomez, Mary Beth Beasley, Maureen Zakowski, Michael Chung, David F. Yankelevitz, Claudia I. Henschke, Emanuela Taioli, Yeqing Zhu, Natela Paksashvili, Lijing Zhang, Lyu Lyu, Huiwen Chan, Jeffrey Zhu, Sydney Kantor, Lauren Lentini, Daniel Nicastri, Ardeshir Hakami-Kermani, Arzu Buyuk, Adie Friedman, Ronald Dreifuss, Stacey Verzosa, Mariya Yakubov, Karina Aloferdova, Patricia Stacey, Simone De Nobrega, Jeffrey Zhu, Sydney Kantor, Lauren Lentini, Ardeshir Hakami-Kermani, Jeffrey Zhu, Sydney Kantor, Lauren Lentini, Raja Flores, Claudia Henschke, Emanuela Taioli, David Yankelevitz, Rebecca Schwartz, Artit Jirapatnakul, Rowena Yip, Huiwen Chan

**Affiliations:** a Department of Diagnostic, Molecular, and Interventional Radiology, Icahn School of Medicine at Mount Sinai, New York, NY, 10029, USA; b Department of Oncology, Centre Hospitalier Universitaire Vaudois (CHUV); Lausanne University, Lausanne, Switzerland; c Department of Thoracic Surgery, Icahn School of Medicine at Mount Sinai, New York, NY, 10029, USA

**Keywords:** Early-stage lung cancer, Octogenarians, Older adults, Quality of life, Lung cancer surgery

## Abstract

**Background:**

As life expectancy increases, more adults aged ≥80 years are diagnosed with early-stage lung cancer. Often these patients are excluded from screening programs and clinical trials due to concerns about comorbidities and surgical risk as evidence on surgical outcomes and quality of life (QoL) remains limited. We aimed to compare postoperative outcomes, survival, and QoL between octogenarians and younger patients undergoing surgery for Stage IA NSCLC.

**Methods:**

We included patients with stage IA non-small cell lung cancer from the Mount Sinai Health System enrolled in the prospective Initiative for Early Lung Cancer Research on Treatment (IELCART) study. Octogenarians (80 years and older) were compared to younger patients in terms of clinical presentation, type of surgery, postoperative outcomes, and survival. Quality of life was assessed using physical and mental health scores at baseline and at 1, 6, and 12 months after surgery. Lung cancer–specific and overall survival were analyzed using Kaplan–Meier methods.

**Findings:**

Among 884 patients, 114 (12.9%) were octogenarians. Compared to 770 younger patients, octogenarians had similar comorbidities but underwent more frequently sublobar resections [90/114 (78.9%) vs. 485/770 (62.4%), *p* = 0.030] and had higher complication rates [46/114 (40%) vs. 168/770 (22%), *p* < 0.0001], particularly cardiovascular. Intensive care unit admissions and readmissions were slightly more frequent. In both age groups, physical and mental health declined at two months but improved by twelve months, with no significant differences. Five-year overall- and lung cancer-specific survival rates were similar between octogenarians and younger patients (overall: 84.2% vs. 87.3%, lung cancer–specific survival: 94.4% vs. 94.5%).

**Interpretation:**

Among octogenarians with early-stage NSCLC, surgical treatment was associated with favorable safety and long-term quality-of-life outcomes in carefully selected patients.

**Funding:**

This study was supported by generous grants from the 10.13039/100000893Simons Foundation (International, Ltd.).

**Research in context**

**Evidence before this study**

We searched PubMed on July 3, 2025, from database inception until the search date, for English-language studies evaluating surgical outcomes in octogenarians with early-stage non-small cell lung cancer (NSCLC), using the terms “octogenarians”, “elderly”, “lung cancer surgery”, “stage I”, “sublobar resection”, “lobectomy”, and “non-small cell lung cancer”. Existing literature suggests that octogenarians are less likely to undergo surgery and more frequently receive non-operative management, often due to concerns about frailty and perioperative risk. Although several retrospective analyses have reported that surgery can be safe and effective in selected older patients, prospective data comparing octogenarians to younger patients with stage IA NSCLC—particularly regarding postoperative recovery, quality of life, and survival—remain scarce.

**Added value of this study**

To our knowledge, this is the first prospective study to directly compare clinical, surgical, and patient-reported outcomes between octogenarians and younger patients undergoing resection for stage IA (≤30 mm) NSCLC. Despite a higher rate of sublobar resections and a slightly increased frequency of postoperative complications, octogenarians had comparable ICU admissions, readmission rates, hospital stays, and long-term survival. Quality of life—both physical and mental—did not significantly differ by age. These findings challenge age-based assumptions and demonstrate that, with appropriate selection, octogenarians can achieve outcomes similar to those of younger patients.

**Implications of all the available evidence**

This study supports the use of curative-intent surgery in well-selected octogenarians with early-stage NSCLC. Chronological age alone should not preclude surgical evaluation. As the proportion of older adults with lung cancer grows, these results highlight the importance of individualized decision-making, incorporating functional status, tumor characteristics, and patient preference, to ensure equitable access to curative treatment.

## Introduction

With rising life expectancy and expanding lung cancer screening programs, an increasing number of older adults are diagnosed with early-stage non-small cell lung cancer (NSCLC), where surgical resection or stereotactic radiotherapy offers the potential for cure.^1^^,^^2^ However, patients aged 80 years and older (octogenarians) remain underrepresented in both screening initiatives and clinical trials due to concerns about comorbidities, surgical risk, and postoperative outcomes.^3^^,^^4^ Consequently, the evidence guiding treatment decisions in this population is limited.

Balancing the potential benefits of curative surgery with the risks of complications and the impact on quality of life (QoL) is particularly critical in this age group. Yet, few studies have explored how surgical resection are tolerated by octogenarians and how it affects QoL in octogenarians compared with younger patients.^5^, ^6^, ^7^

Moreover, most existing studies predate the recent phase 3 trials that established lung-sparing surgery as standard for small cancers and do not specifically focus on stage IA NSCLC, further limiting our understanding of patient outcomes after sublobar resection of stage 1A disease.^8^^,^^9^

We hypothesize that with appropriate selection, curative-intent surgery is well tolerated by octogenarians and may offer similar benefits in terms of recovery and QoL. For this analysis, we used the multi-institutional study cohort from the Initiative for Early Lung Cancer Research on Treatment (IELCART) cohort, which is a prospectively enrolled first primary early-stage lung cancer cohort of patients.^10^

In this study, we compared octogenarians to younger patients undergoing resection for stage IA NSCLC at the Mount Sinai Health System (MSHS), assessing the prevalence, clinical characteristics, surgical outcomes, postoperative complications, quality of life, and survival.

## Methods

We conducted our study using patients prospectively enrolled in IELCART. Patients were eligible for inclusion in this report if they had first primary stage IA non-small cell lung cancer (NSCLC) measuring ≤30 mm on preoperative computed tomography (CT) and underwent surgical resection at the MSHS between 2016 and December 2024. All participants provided informed consent to undergo low-dose CT screening as part of a HIPAA-compliant, IRB-approved cohort study (Mount Sinai, STUDY-12-00212) and the principles of the Declaration of Helsinki.

During the enrollment process for IELCART, we gathered baseline demographic information for each patient, which included age, sex, and education level (categorized as less than a college degree or college degree or higher). Smoking status (current, former, never smoker), pack-years of cigarette smoking, and 12 different self-reported comorbidities—presence of asthma, emphysema or chronic obstructive pulmonary disease, high blood pressure, high cholesterol, angioplasty or stent, myocardial infarction, stroke, peripheral vascular disease, liver disease, diabetes, kidney disease and history of cancers other than lung—were also documented. Height and weight were documented as was body mass index (BMI) in kg/m^2^. Patient race (White, African American or Black, Asian, and other) and ethnicity (Hispanic *vs.* non-Hispanic) were documented. Pre-surgical CT scan findings including nodule consistency documented as solid, part-solid, or nonsolid according to published criteria^11^ and post-surgical pathologic findings including tumor characteristics, surgical details, postoperative complications, and length of stay were documented.

Operative complications occurring in the immediate inpatient setting were identified based on data from the Society of Thoracic Surgeons General Thoracic Surgery Database (STS-GTSD).^12^ These complications were routinely ascertained by trained data abstractors through comprehensive review of the medical record. Pulmonary events included pneumonia, acute respiratory distress syndrome (ARDS), pulmonary embolism, prolonged initial ventilation exceeding 48 h, pleural effusion requiring drainage, respiratory failure, and pneumothorax requiring chest tube reinsertion, along with other pulmonary complications. Cardiovascular complications comprised atrial and ventricular arrhythmias, myocardial infarction, deep venous thrombosis, and other cardiovascular events. Additional complications included gastrointestinal issues such as ileus; requiring blood transfusion; urologic complications comprised of urinary tract infection, urinary retention, and discharge with a Foley catheter; and infections such as empyema, pulmonary infection, and sepsis. Neurologic complications included new central neurological events and delirium. Other miscellaneous complications were also captured.

During the pre-treatment clinic visit, interviews were conducted to collect patients’ pre-treatment QoL scores. If patients were not available for in-person interviews, telephone interviews or mailed questionnaires were completed by patients. QoL scores were collected during follow-up clinic visits scheduled at 1, 6, and 12 months postoperatively, and annually thereafter. Quality of life (QoL) was evaluated using the validated 12-item Short Form Survey Version 2(SF-12v2), which includes physical (PCS) and mental (MCS) component scores.^13^ These instruments reflect eight health domains: physical functioning, role limitations due to physical health, bodily pain, general health perceptions, vitality, social functioning, role limitations due to emotional problems, and mental health; all within the context of the past 4 weeks. The norm-based PCS and MCS scores for the general United States population have a mean of 50 and a standard deviation of 10, with higher scores indicating better physical and mental health. A difference of 3 points is considered clinically significant.^7^

Lung cancer–specific survival and overall survival served as the primary endpoints. Participants diagnosed with NSCLC were systematically followed at least once per year by site investigators and study coordinators at each institution. Follow-up information was reported to the central coordinating center in line with study requirements. The date and cause of death were obtained from treating physicians, family members, or both, and were further validated using data from the National Death Index. Any death occurring within 30 days of surgical intervention or other lung cancer–directed therapy was categorized as a lung cancer–related death, with deaths from other causes treated as competing events. Follow-up time was calculated as the time in months between the date of surgery and either the date of death or March 31, 2025, whichever occurred first.

### Statistical analysis

Characteristics of patients aged ≥80 years (octogenarians) were compared to those aged <80 years (younger patients). Continuous data were summarized as means (SD) or medians (IQR), and categorical data as frequencies (%). For univariate analyses, associations among categorical variables were assessed using the Chi-square test, with Fisher's exact test applied when necessary. The unpaired t-test compared means for normally distributed data; otherwise, the Mann–Whitney U test was used. When comparing more than two groups, the Kruskal–Wallis test was utilized.

Longitudinal changes in QoL were modeled using piecewise linear mixed-effects (LMMs), incorporating fixed effects for age group and time since surgery (in months), along with random effects to account for within-subject correlations over time. An inflection point at 2 months postoperatively was included to capture early recovery, with separate slopes estimated for two time intervals: baseline to 2 months, and 2.1–12 months after surgery.^7^ Models were adjusted for relevant covariates, including sex, smoking status, and pack-years, comorbidity, maximum nodule diameter on CT, nodule consistency, histology, extent of resection, and surgical approach. Model assumptions (linearity, normality of residuals and random effects, homoscedasticity, and within-subject correlation structure) were evaluated using diagnostics plots and statistical tests. In the presence of heteroscedasticity or residual correlation, cluster-robust standard errors were applied and sensitivity analyses confirmed robustness of results. Estimated marginal means of QoL scores were derived to compare QoL trajectories between octogenarians and younger patients. Patterns of missing QoL data were examined to assess plausibility of missing completely at random or missing at random. Under these assumptions, mixed-effects models using maximum likelihood without imputation were applied.

Overall survival was analyzed using Cox proportional hazards regression adjusted for sex, smoking status, pack-years, nodule size, nodule consistency, surgical extent, COPD, and histology. Lung cancer-specific mortality was evaluated using Cox proportional hazard model and Fine–Gray competing risk models with non-cancer death as a competing event. Hazard ratios (HRs) and subdistribution hazard ratios (SHRs) with 95% CIs were reported. Statistical significance was defined as *p* < 0.05 (two-sided). All statistical analyses were performed using R version 4.2.2 statistical software (R Foundation for Statistical Computing, Vienna, Austria).

### Role of the funding source

The funder of the study had no role in study design, data collection, data analysis, data interpretation, or writing of the report.

## Results

**Fig. 1:**
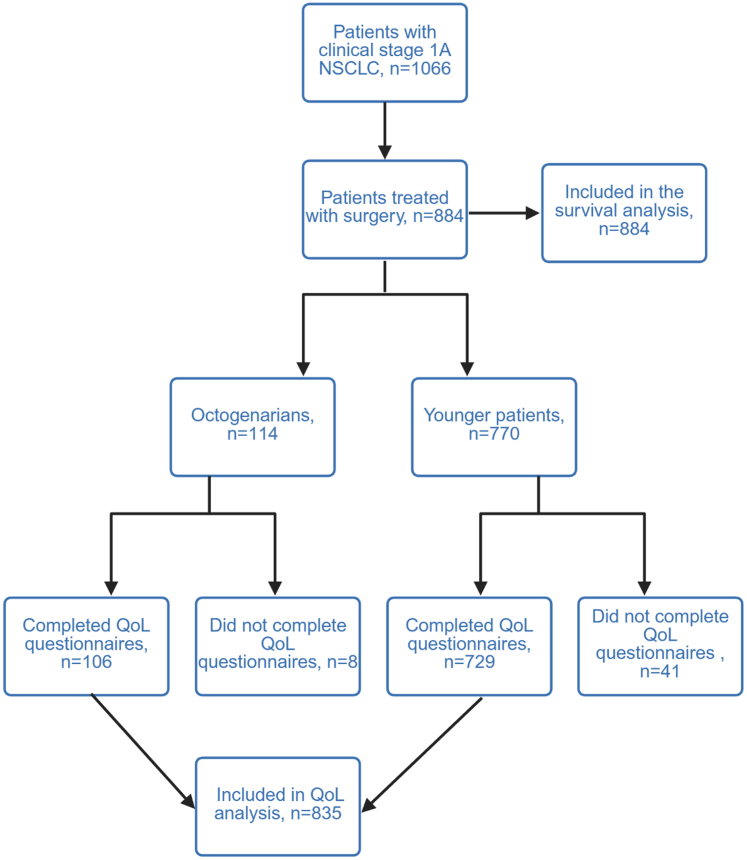
CONSORT flow diagram of participant selection and study inclusion.

We identified 884 patients who underwent surgery for clinical stage IA (≤30 mm) NSCLCs (Fig. 1). Of these, 114 (13%) were aged ≥80 years (Table 1). Median age in octogenarians compared with younger patients was 82 years (IQR 81–85) vs. 69 years (IQR 63–74) (*p* < 0.0001).

**Table 1:** Baseline characteristics and self-reported comorbidities in patients aged <80 versus ≥80 years.

	Overall N = 884^a^	<80 Years N = 770^a^	≥80 Years N = 114^a^	*p*-Value^b^
**Age, years, median (IQR)**	70 (64–76)	69 (63–74)	82 (81–85)	<0.0001
**Sex**				0.011
Female	538 (61%)	481 (62%)	57 (50%)	
Male	346 (39%)	289 (38%)	57 (50%)	
**Race**				0.0005
White	545 (62%)	454 (59%)	91 (80%)	
Black or African American	134 (15%)	124 (16%)	10 (8.8%)	
Asian	100 (11%)	93 (12%)	7 (6.1%)	
Others	101 (11%)	95 (12%)	6 (5.3%)	
**Ethnicity**				0.12
Hispanic or Latino	136 (15%)	124 (16%)	12 (11%)	
Not Hispanic or Latino	748 (85%)	646 (84%)	102 (89%)	
Smoking status				0.0012
Current	106 (12%)	103 (13%)	3 (2.6%)	
Former	533 (60%)	450 (58%)	83 (73%)	
Never	245 (28%)	217 (28%)	28 (25%)	
Pack-years among smokers, median (IQR)	29 (13–48)	29 (13–47)	30 (15–50)	0.50
Cardiac	118 (13%)	98 (13%)	20 (18%)	0.16
Hypertension	463 (52%)	397 (52%)	66 (58%)	0.21
COPD^c^	187 (21%)	176 (23%)	11 (9.6%)	0.0012
Diabetes	173 (20%)	155 (20%)	18 (16%)	0.28
BMI^d^, median (IQR)	26.2 (23.0–30.2)	26.4 (23.1–30.4)	25.1 (22.8–28.2)	0.018
Pulmonary function^c^:				
FEV1^e^ % predicted		88 (76–101)	94 (82–109)	0.0003
DLCO^f^ % predicted		78 (65–90)	73 (65–86)	0.23
Family history of lung cancer				0.27
None	719 (81%)	622 (81%)	97 (85%)	
Yes	165 (19%)	148 (19%)	17 (15%)	

a Median, IQR: Interquartile range (Q1–Q3); n (%).

b Wilcoxon rank sum test; Pearson's Chi-squared test.

c COPD: Chronic obstructive pulmonary disease.

d BMI: Body mass index.

e FEV_1_ % predicted: forced expiratory volume in 1 s expressed as a percentage of the predicted value.

f DLCO % predicted: diffusing capacity of the lung for carbon monoxide expressed as a percentage of the predicted value.

### Clinical presentation and tumor features of octogenarians with lung cancer compared with younger patients

Among octogenarians, the sex distribution was equal between male and female patients (50% [57/114] vs. 50% [57/114]), whereas females patients were predominant among younger participants (62% [481/770] vs. 38%, [289/770] *p* = 0.011). Race also varied significantly between age groups, with octogenarians being more frequently White (80% [91/114] vs. 59% [454/770]), and less frequently Black or African American (8.8% [10/114] vs. 16% [124/770]), Asian (6.1% [7/114] vs. 12% [93/770]), and Other (5.3% [6/114] vs. 12% [95/770]) (*p* = 0.0005).

Smoking history differed by age (*p* = 0.0012), with fewer octogenarians still currently smoking (2.6% [3/114] vs. 13% [103/770]). The frequency of patients who had never smoked was similar (25% [28/114] vs. 28% [217/770]) for both groups. Self-reported comorbidities, including hypertension (58% [66/114] vs. 52% [397/770], *p* = 0.21) and diabetes (16% [18/114] vs. 20% [155/770], *p* = 0.28), were similar, though COPD was significantly lower in octogenarians (9.6% [11/114] vs. 23% [176/770], *p* = 0.012). Octogenarians had a significantly lower BMI compared to younger patients (25.1 vs. 26.4; *p* = 0.018). Pulmonary function differed between the groups, with octogenarians showing higher FEV_1_ percent predicted (94% vs. 88%, *p* = 0.0003), while DLCO percent predicted was similar (73% vs. 78% *p* = 0.23). Family history of lung cancer was comparable (15% [17/114] vs. 19% [148/770], *p* = 0.27) (Table 1).

Nodule consistency on CT, median nodule diameter and location were not significantly different (Table 2). Surgical approaches were similar in both groups (*p* = 0.11). However, octogenarians underwent fewer lobectomies (21% [24/114] vs. 35% [272/770]), more wedge resections (71% [81/114] vs. 56% [435/770]) and segmentectomies (8% [9/114] vs. 6.4% [50/770]) (*p* = 0.030). Resected tumors were larger in octogenarians (median 18 mm vs. 16 mm, *p* = 0.014). Histological classifications differed significantly (*p* = 0.0032), with octogenarians having more adenocarcinomas (88% [100/114] vs. 75% [576/770]) and fewer squamous cell carcinomas (8.8% [10/114] vs. 10% [80/770]).

**Table 2:** Comparison of primary tumor characteristics between patients aged <80 and ≥ 80 years.

Characteristic	<80 Years N = 770^a^	≥80 Years N = 114^a^	*p*-Value^b^
**Nodule consistency**			0.58
Nonsolid	50 (6.5%)	6 (5.3%)	
Part-solid	127 (17%)	23 (20%)	
Solid	593 (77%)	85 (75%)	
**Pathological stage**			0.53
0	7 (0.9%)	1 (0.9%)	
IA	590 (79%)	86 (77%)	
IB	93 (12%)	17 (15%)	
IIA	18 (2.4%)	0 (0%)	
IIB	21 (2.8%)	4 (3.6%)	
IIIA	13 (1.7%)	3 (2.7%)	
IVA	5 (0.7%)	0 (0%)	
**Nodule size (mm), median (IQR)**	15 (10–20)	16 (11–21)	0.31
**Nodule location**			1.00
Upper/Middle lobes	509 (66%)	76 (67%)	
Lower lobes	257 (33%)	38 (33%)	
Other	4 (0.5%)	0 (0%)	
**Surgery approach**			0.11
Robotic-assisted	139 (18%)	14 (12%)	
Thoracotomy	125 (16%)	14 (12%)	
Video-assisted thoracoscopic surgery	504 (66%)	86 (75%)	
**Surgical extent**			0.030
Bilobectomy	11 (1.3%)	0 (0%)	
Lobectomy	272 (35.3%)	24 (21%)	
Pneumonectomy	2 (0.3%)	0 (0%)	
Segmentectomy	50 (6.5%)	9 (7.9%)	
Wedge Resection	435 (56%)	81 (71%)	
**Size of resected tumors (mm)**	16 (12–22)	18 (15–25)	0.014
**Histology**			0.0032
Adenocarcinoma	576 (75%)	100 (88%)	
Squamous cell	80 (10%)	10 (8.8%)	
Carcinoid^c^	93 (12%)	3 (2.6%)	
Other^d^	21 (2.7%)	1 (0.9%)	
**Angiolymphatic invasion**	221 (29%)	46 (40%)	0.012
**Vascular invasion**	29 (3.8%)	4 (3.5%)	1.00
**Margin (mm)**	15 (8–25)	15 (10–22)	0.53
**Margin-to-tumor ratio**	0.95 (0.47–1.54)	0.83 (0.54–1.23)	0.58
**Lymph nodes resected**	2.00 (1.00, 4.00)	2.00 (1.00, 4.00)	0.15
**Lymph nodes positive**			0.67
0	731 (94.94%)	107 (93.86%)	
1	26 (3.38%)	4 (3.51%)	
2	6 (0.78%)	1 (0.88%)	
≥3	7 (0.91%)	2 (1.75%)	

a n (%); Median (Q1 - Q3).

b Pearson's Chi-squared test; Wilcoxon rank sum test; Fisher's exact test.

c Carcinoid includes 89 typical and 7 atypical.

d Other histology includes adenosquamous, large cell.

Octogenarians more frequently had angiolymphatic invasion (40% [46/114] vs. 29% [221/770], *p* = 0.012), whereas vascular invasion (3.5% [4/114] vs. 3.8% [29/770]) and margin status (15 mm vs. 15 mm, *p* = 0.53) did not differ significantly, nor did the margin-to-tumor ratio (0.83 vs. 0.83, *p* = 0.58). The distribution of pathological stage was similar between the two groups, as were the number of resected lymph nodes (2 vs. 2, *p* = 0.15) and the proportion of patients with lymph node involvement, with the majority being node-negative in both groups (93.9% [107/114] vs. 94.9% [731/770]).

### Comparison of postoperative complications in octogenarians and younger patients

Octogenarians had similar postoperative hospital stay compared with younger patients (median 3 days, *p* = 0.10), but significantly more complications (40% [46/114] vs. 22% [168/770], *p* < 0.0001), particularly urologic complications mainly due to urinary catheter use (28% [32/114] vs. 11.7% [90/770], *p* < 0.0001), neurologic events (5.3% [6/114] vs. 0.9% [7/770], *p* = 0.0032), and cardiovascular events (11% [12/114] vs. 5.8% [45/770], *p* = 0.06) (Table 3). Pulmonary (8.8% [10/114] vs. 5.8% [45/770], *p* = 0.23) and infectious complications (1.8% [2/114] vs. 0.8% [6/770], *p* = 0.28), intensive care unit admissions (2.6% [3/114] vs. 0.8% [6/770]), and 30-day readmissions (7.0% [8/114] vs. 3.8% [29/770]) were also slightly more frequent in octogenarians, although these differences were not statistically significant. Within 30 days of surgery, no death occurred. Within 90-days of surgery, 3 deaths occurred (2 from the younger group and 1 from the octogenarians).

**Table 3:** Postoperative outcomes and complications in patients aged <80 versus ≥80 years.

Characteristic	Overall N = 884^a^	<80 Years N = 770^a^	≥80 Years N = 114^a^	*p*-Value^b^
**Length of stay (Days)**	3.00 (2.00, 4.00)	3.00 (2.00, 4.00)	3.00 (3.00, 4.00)	0.10
**ICU**^c^**Care required**	9 (1.0%)	6 (0.8%)	3 (2.6%)	0.10
**Postoperative complications**	214 (24%)	168 (22%)	46 (40.4%)	<0.0001
**Pulmonary**	55 (6.2%)	45 (5.8%)	10 (8.8%)	0.23
Air leak greater than 5 days duration	38 (4.3%)	30 (3.9%)	8 (7.0%)	>0.9
Pneumothorax req. CT^d^ reinsertion	4 (1.9%)	4 (0.5%)	0 (0%)	0.58
Other pulmonary event	11 (5.2%)	9 (0.1%)	2 (0.2%)	1.00
**Cardiovascular**	57 (6.4%)	45 (5.8%)	12 (11%)	0.06
Atrial arrhythmia req. treatment	51 (5.8%)	40 (5.2%)	11 (9.6%)	1.00
Ventricular arrhythmia req. treatment	4 (0.5%)	4 (0.5%)	0 (0%)	0.58
Other CV^e^ event	3 (0.3%)	1 (0.1%)	2 (4.3%)	0.12
**Gastrointestinal**	4 (0.5%)	2 (0.3%)	2 (1.8%)	0.08
Ileus	1 (0.1%)	0 (0%)	1 (1.8%)	0.22
**Hematology**	7 (0.8%)	6 (0.8%)	1 (0.9%)	1.00
Blood transfusion	7 (0.8%)	6 (0.8%)	1 (0.9%)	1.00
**Urologic**	122 (13%)	90 (12%)	32 (28%)	<0.0001
Urinary tract infection	11 (1.2%)	7 (0.9%)	4 (3.5%)	0.26
Urinary retention req. catherization	113 (13%)	83 (11%)	30 (26%)	0.06
Discharged with Foley catheter	23 (2.6%)	16 (2.1%)	7 (6.1%)	0.29
**Neurology**	13 (1.5%)	7 (0.9%)	6 (5.3%)	0.0032
New central neurological event	1 (0.5%)	1 (0.1%)	0 (0%)	1.00
Delirium	12 (1.4%)	6 (0.8%)	6 (5.3%)	0.025
**Infection**				
Chylothorax req. medical intervention	3 (0.3%)	3 (0.4%)	0 (0%)	1.00
**Miscellaneous**	6 (0.7%)	6 (0.8%)	0 (0%)	1.00
**30-day readmission after surgery**	37 (4.2%)	29 (3.8%)	8 (7.0%)	0.13

a Median (Q1, Q3); n (%).

b Wilcoxon rank sum test; Fisher's exact test; Pearson's Chi-squared test.

c ICU: Intensive care unit.

d CT: Computed tomography.

e CV: Cardiovascular.

### Longitudinal quality of life assessment in octogenarians and younger adults after lung cancer surgery

Among the 884 patients, QoL data was available in 835 (94.5%) patients. QoL questionnaire completion rates (Supplementary Table S1) were highest at 1 month post-surgery (83% in both age groups) and lower at other timepoints (56–65%). Completion rates did not differ significantly between octogenarians and non-octogenarians at any timepoint (all *p* > 0.05).

**Fig. 2:**
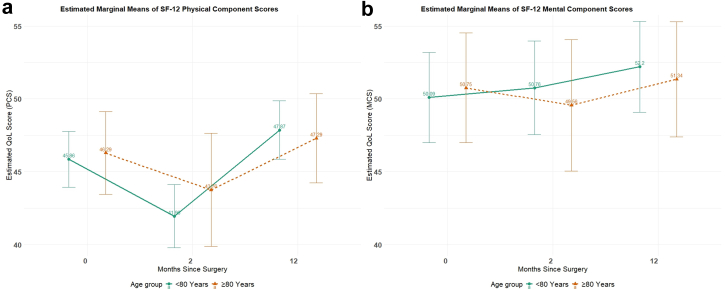
a: Estimated marginal means of the physical component summary (PCS) at baseline and 2 and 12 months after surgery in octogenarians and younger patients. b: Estimated marginal means of the mental component summary (MCS) at baseline and 2 and 12 months after surgery in octogenarians and younger patients.

At baseline, octogenarians had an estimated physical component summary (PCS) score of 46.29 (95% CI: 43.45, 49.13) compared to 45.86 (95% CI: 43.95, 47.77) for younger patients (Fig. 2a). At 2 months post-surgery, both groups showed decline in PCS scores: 43.76 (95% CI: 39.88, 47.64) in octogenarians and 41.96 (95% CI: 39.80, 44.12) in younger patients. By 12 months after surgery, both age groups showed recovery, with octogenarians scoring 47.29 (95% CI: 44.23, 50.35) and younger patients scoring 47.87 (95% CI: 45.87, 49.86).

For the Mental Component Summary (MCS), octogenarians had an estimated baseline score of 50.75 (95% CI: 46.57, 54.94) compared to 50.09 (95% CI: 46.36, 53.83) in younger patients (Fig. 2b). At 2 months, the MCS score declined to 49.56 (95% CI: 44.78, 54.34) in octogenarians, while remaining stable at 50.76 (95% CI: 47.04, 54.48) in younger patients. By 12 months post-surgery, MCS scores had improved in both groups to 51.34 (95% CI: 46.57, 56.11) in octogenarians and 52.20 (95% CI: 48.46, 55.95) in younger patients.

Pairwise comparisons (Table 4) showed no statistically significant differences between age groups at any time point for either PCS (baseline: *p* = 0.70; 2-month: *p* = 0.33; 12-month: *p* = 0.68) or MCS (baseline: *p* = 0.51; 2-month: *p* = 0.46; 12-month: *p* = 0.56). The absolute differences between groups were small (all <2 points), below the threshold of clinical significance (typically considered 3 points for SF-12 scores).

**Table 4:** Adjusted least-squares mean quality-of-life scores in patients aged <80 and ≥ 80 years based on a linear piecewise mixed-effects model.

	Baseline estimated score			2-Month estimated score			12-Month estimated score		
	Estimate (95% CI)	SE	*p*-Value^c^	Estimate (95% CI)	SE	*p*-Value^c^	Estimate (95% CI)	SE	*p*-Value^c^
PCS^a^(SF-12)^b^									
<80 Group	45.86 (43.95, 47.77)	0.97	–	41.96 (39.80, 44.12)	1.10	–	47.87 (45.87, 49.86)	1.02	–
≥80 Group	46.29 (43.45, 49.13)	1.45	–	43.76 (39.88, 47.64)	1.98	–	47.29 (44.23, 50.35)	1.56	–
Difference	0.43	1.12	0.70	1.80	1.85	0.33	−0.57	1.35	0.68
MCS^d^(SF-12)									
<80 Group	50.09 (46.36, 53.83)	1.90	–	50.76 (47.04, 54.48)	1.90	–	52.20 (48.46, 55.95)	1.76	–
≥80 Group	50.75 (46.57, 54.94)	2.13	–	49.56 (44.78, 54.34)	2.44	–	51.34 (46.57, 56.11)		–
Difference	0.66	0.99	0.51	−1.20	1.61	0.46	−0.86	0.56	0.56

a PCS – Physical component summary.

b SF-12 – 12-Item Short Form Health survey (SF-12).

c Adjusted for sex, smoking status and pack-years, COPD, maximum nodule size on CT, nodule consistency, histology and surgery extent.

d MCS – Mental component summary.

### Overall and lung cancer-specific survival

**Fig. 3:**
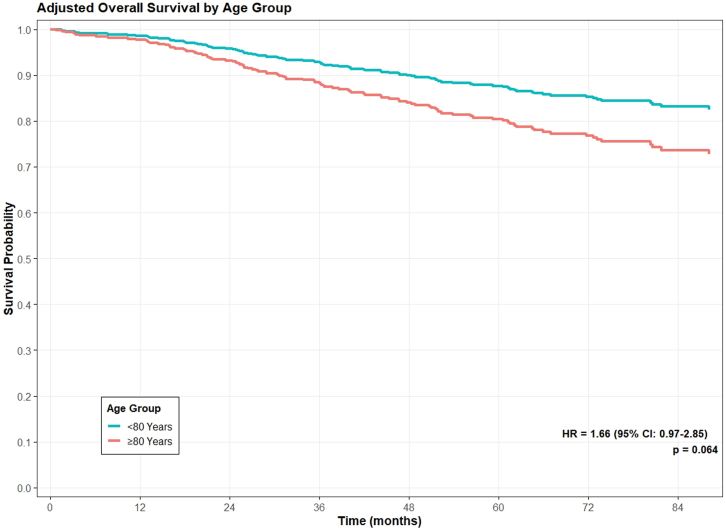
Adjusted overall survival in octogenarians (n = 114) and younger patients (n = 770).

**Fig. 4:**
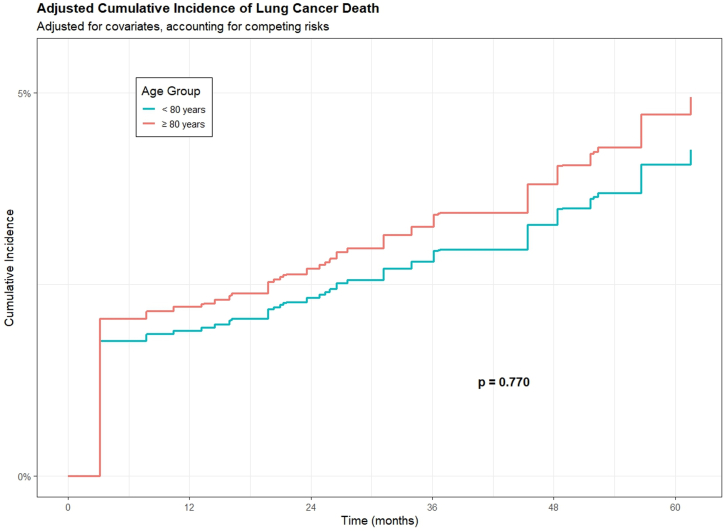
Adjusted cumulative incidence of lung cancer–specific death in octogenarians (n = 114) and younger patients (n = 770).

All 884 patients were included in the survival analysis. The median follow-up from surgery to death or last contact was 4.28 years (interquartile range: 2.12–6.54). During this period, 103 patients died, including 37 from lung cancer. The 5-year overall survival rate was slightly lower in octogenarians compared to younger patients (84.2% vs. 87.3%), but this difference was not statistically significant (Fig. 3, *p* = 0.10). Five-year lung cancer–specific survival rates were comparable between octogenarians and younger individuals (94.4% vs. 94.5%; Fig. 4, *p* = 0.87), with both groups demonstrating high lung cancer-specific survival.

After adjusting for clinical covariates, there was no statistically significant difference in overall mortality between octogenarians and younger patients (adjusted HR 1.66, 95% CI: 0.97–2.85, *p* = 0.06) (Table 5, Fig. 3). For lung cancer-specific mortality, competing risk analysis accounting for non-cancer deaths showed no significant difference between age groups (subdistribution HR 1.16, 95% CI: 0.43–3.12, *p* = 0.77) (Table 6, Fig. 4).

**Table 5:** Model output of the adjusted overall survival Cox model.

Variables	HR (95%CI)^a^	*p*-Value
**Age group**		
< 80 Years	REF	
≥80 Years	1.66 (0.97–2.85)	0.06
**Sex**		
Female	REF	
Male	1.27 (0.85–1.90)	0.25
**Smoking status**		
Never smokers	REF	
Former smokers	0.93 (0.49–1.77)	0.84
Current smokers	1.11 (0.50–2.44)	0.80
**Pack years of smokers**	1.01 (1.00–1.01)	0.20
**Nodule size**	1.03 (0.99–1.06)	0.12
**Nodule consistency**		
Nonsolid	REF	
Part-solid	3.77 (0.47–30.10)	0.21
Solid	8.04 (1.11–58.32)	0.039
**Surgery extent**		
Sublobar	REF	
Lobectomy+	0.81 (0.53–1.25)	0.35
**COPD**^b^	1.70 (1.07–2.69)	0.02
**Histology**		
Adenocarcinoma	REF	
Squamous cell	1.29 (0.76–2.19)	0.34
Carcinoid	0.17 (0.04–0.72)	0.016
Other	2.10 (0.89–4.96)	0.09

a HR: Hazard ratio; CI: Confidence interval.

b COPD: Chronic obstructive pulmonary disease.

**Table 6:** Estimation of risk of lung cancer death using cause-specific hazard model and fine–gray competing risk model.

Variables	HR (95%CI)^a^ of cause specific hazard model	*p*-Value	HR (95%CI)^a^ of Fine–gray competing risk model	*p*-Value
**Age group**				
< 80 Years	REF		REF	
≥80 Years	1.18 (0.43–3.18)	0.75	1.16 (0.43–3.12)	0.77
**Sex**				
Female	REF		REF	
Male	0.75 (0.38–1.51)	0.42	0.75 (0.39–1.47)	0.41
**Smoking status**				
Never smokers	REF		REF	
Former smokers	2.64 (0.71–9.82)	0.15	2.66 (0.70–10.06)	0.15
Current smokers	3.33 (0.73–15.14)	0.12	3.29 (0.71–15.17)	0.13
**Pack-years of smoking**	1.00 (0.99–1.02)	0.71	1.00 (0.98–1.02)	0.78
**Nodule Size (mm)**	1.05 (0.99–1.11)	0.08	1.05 (0.99–1.11)	0.10
**Nodule consistency**				
Nonsolid/part-solid	REF		REF	
Solid	4.92 (1.13–21.34)	0.03	4.91 (1.17–20.57)	0.03
**Surgery extent**				
Sublobar	REF		REF	
Lobectomy	0.44 (0.20–0.97)	0.04	0.44 (0.19–1.00)	0.05
**COPD**^b^	2.11 (1.02–4.36)	0.04	2.08 (0.99–4.38)	0.05
**Histology**				
Adenocarcinoma	REF		REF	
Squamous cell	0.50 (0.17–1.45)	0.20	0.51 (0.17–1.47)	0.21
Carcinoid/other	0.39 (0.09–1.65)	0.20	0.39 (0.09–1.71)	0.21

a HR: Hazard ratio; CI: Confidence interval.

b COPD: Chronic obstructive pulmonary disease.

## Discussion

Our study of 884 prospectively enrolled IELCART patients compared clinical and surgical outcomes of 114 (12.9%) octogenarians and 770 (87.1%) younger patients with a first primary stage IA (≤30 mm) lung cancer. Octogenarians were more frequently white and had a higher proportion of male patients, had similar comorbidities to younger patients, though COPD was less frequent, possibly due to survivor bias or stricter selection.^14^ Tumor and nodule characteristics were comparable between groups, although octogenarians more frequently had adenocarcinoma and angiolymphatic invasion, and slightly larger resected tumors—possibly reflecting differences in smoking history, referral bias, or differences in tumor biology.^15^^,^^16^

Surgical extent differed by age, with octogenarians more commonly undergoing sublobar resection. Despite this, postoperative recovery was broadly similar between groups. Octogenarians experienced more complications, but had similar ICU admissions, readmissions, and hospital stays, suggesting recovery can match that of younger patients with appropriate perioperative care in carefully selected octogenarians.

Longitudinal assessments showed no significant differences in physical or mental quality of life between the two groups. Both experienced a decline in physical function at two months, with recovery to baseline by 12 months. Mental health remained stable. These findings suggest well-selected octogenarians can undergo surgery without significant long-term impact on quality of life, consistent with prior studies showing postoperative improvement following an early decline.^7^^,^^17^

Despite growing interest in the care of octogenarians, to our knowledge, this is the first study focused exclusively on patients with first primary clinical stage IA lung cancer.^18^^,^^19^ As in prior work, octogenarians had survival and quality of life outcomes comparable to younger patients.^5^ Five-year overall and lung cancer–specific survival were similar, supporting the feasibility of curative surgery in well-selected older adults. Notably, these outcomes were achieved despite more limited surgical resections.

Our results support surgical management of small lung tumors in fit octogenarians and suggest they may also benefit from screening, despite being excluded under current USPSTF criteria.^20^ These findings are consistent with prior studies, including the JACS1303 trial, which reported low operative and hospital mortality (1.0% and 1.6%) in selected octogenarians.^21^^,^^22^ Other retrospective studies also found no significant differences in survival or postoperative mortality between octogenarians and those aged 70–79 years, reinforcing that age alone should not guide treatment decisions.^23^^,^^24^

National data may offer a more nuanced picture. In a U.S. cohort of over 22,000 patients with stage IA NSCLC, octogenarians had significantly higher in-hospital (2.4% vs. 0.9%), 90-day (7.2% vs. 2.9%), and one-year mortality (15.2% vs. 7.3%) compared with patients aged 65–69.^3^ It is worth noting that our study used a different age comparison (≥80 vs. <80) and included longer-term follow-up (median 4.5 years). The more favorable outcomes observed in our cohort likely reflect differences in patient selection, patient volume and care settings, and surgical practices.

Overall, our findings support the growing view that curative surgery is safe and effective in well-selected octogenarians. Limited resection may offer similar recovery and quality of life to that of younger patients. Surgical decisions should be based on functional status, comorbidities, and patient preferences, rather than age alone, since chronological and physiological age often diverge.^3^^,^^25^, ^26^, ^27^ Broader inclusion of very elderly patients in prospective studies is needed to better reflect real-world populations. In the study by Altorki et al., a 90-day mortality of just 1.4% was reported after resection for stage IA NSCLC, yet only 4.6% of their cohort was over 80 years of age.^9^ Further research is needed to improve risk stratification and assess non-surgical options like stereotactic body radiation therapy for high-risk surgical candidates.^10^

Our study has limitations. Although 884 patients with stage IA tumors ≤3 cm were enrolled since 2016, only 12.9% (n = 114) were octogenarians, limiting subgroup analysis. All patients were treated within a single health system, which may affect generalizability, although the cohort spanned seven hospitals and nine surgeons.^28^^,^^29^ As only surgical candidates were included, octogenarians likely represent a healthier subset, introducing selection bias. Functional status was derived from medical records and may not fully capture frailty. Standardized complication grading (e.g., Clavien–Dindo) was not captured in IELCART and was therefore unavailable.^30^

### Conclusions

Among 884 patients with resected lung cancers ≤30 mm, 12.9% (n = 114) were octogenarians. These octogenarians had similar comorbidities and tumor characteristics but more frequently had sublobar resections. Although postoperative complications were slightly more common, hospital stay and short-term recovery were similar across age groups. Physical health and lung cancer symptoms initially declined but improved over time, with patterns comparable between age groups. Notably, 5-year overall and lung cancer–specific survival rates were comparable, supporting the feasibility of curative surgery in well-selected older adults and challenging the notion that age alone should preclude surgery. Further research is needed to refine risk stratification and treatment strategies for this growing, underrepresented population.

## Contributors

L. Gros was involved in the study conception and design, data interpretation, and writing & editing of the original draft. R. Yip was involved in the study design, administrative support, provision of study materials, collection & assembly of data, data analysis, data interpretation, supervision, writing & editing of the original draft, and provided critical approval of the manuscript. W. Ma and J. Zhang were involved in the collection & assembly of data, data analysis, data interpretation, writing & editing of the original draft, and provided critical approval of the manuscript. J. Zhu was involved in the study design, collection & assembly of data, data interpretation, writing & editing of the original draft, and provided critical approval of the manuscript. S. Kantor and S. Cai were involved in the collection & assembly of data and provided critical approval of the manuscript. A.J. Kaufman, A.S. Wolf, A. Hakami-Kermani, D. Nicastri, D.S. Lee, K.J. Song, B. Housman provided critical approval of the manuscript. D.F. Yankelevitz and C.I. Henschke were involved in the study design, administrative support, provision of study materials, collection & assembly of data, data interpretation, supervision, writing & editing of the original draft, and provided critical approval of the manuscript. E. Taioli was involved in administrative support and provided critical approval of the manuscript. R.M. Flores was involved in the administrative support, provision of study materials, data interpretation, supervision, and provided critical approval of the manuscript.

## Data sharing statement

Individual participant data will not be made available. The study protocol is available upon request.

## Declaration of interests

• Dr. Yankelevitz is a named inventor on a number of patents and patent applications related to the evaluation of chest diseases including measurements of chest nodules. Dr. Yankelevitz has received financial compensation for the licensing of these patents. In addition, he is a consultant and co-owner of Accumetra, a private company developing tools to improve the quality of CT imaging. He is on the advisory board and owns equity in HeartLung, a company that develops software related to CT scans of the chest. He is on the medical advisory board of Median Technology that is developing technology related to analyzing pulmonary nodules and is on the medical advisory board of Carestream, a company that develops radiography equipment. He is also on the advisory board for LungLife AI.
• Dr. Claudia Henschke is also an inventor of the patents and pending patents owned by Cornell Research Foundation (CRF). As of April 2009, she has divested herself of all royalties and other interests arising from these. She is on the medical advisory board for LungLife AI.
• Others: None

## Appendix A Supplementary data

Supplementary Table

Investigators
