# Supplementary material for: Surgical outcomes and quality of life in octogenarians with early-stage non-small cell lung cancer: a prospective cohort study
Source: Lancet Reg Health Am. 2026 Mar 13;56:101428. doi: 10.1016/j.lana.2026.101428 (PMC13000533; doi:10.1016/j.lana.2026.101428)
Supplement: Supplementary Table [file mmc1.docx]

**Supplementary Table 1: QoL Questionnaire Completion Rates by Age Group (835)**

| Timepoint | Octogenarians n/N (%) | Non-octogenarians n/N (%) | p-value |
| --- | --- | --- | --- |
| Baseline | 59/106 (55.7%) | 424/729 (58.2%) | 0.702 |
| 1 month | 88/106 (83%) | 607/729 (83.3%) | 1.000 |
| 6 months | 59/106 (55.7%) | 470/729 (64.5%) | 0.099 |
| 12 months | 62/106 (58.5%) | 442/729 (60.6%) | 0.753 |
