# Supplementary material for: Surgical outcomes and quality of life in octogenarians with early-stage non-small cell lung cancer: a prospective cohort study
Source: Lancet Reg Health Am. 2026 Mar 13;56:101428. doi: 10.1016/j.lana.2026.101428 (PMC13000533; doi:10.1016/j.lana.2026.101428)
Supplement: Investigators [file mmc2.docx]

**Surgical Outcomes and Quality of Life in Octogenarians With Early-Stage Non-Small Cell Lung Cancer: A Prospective Cohort Study**

Louis Gros^a,b,d^, Rowena Yip^a,d^, Wenchao Ma^a^, Jeffrey Zhu^a^, Jiafang Zhang^a^, Sydney Kantor^a^ Siyang Cai^a^, Andrew J Kaufman^c^, Andrea S Wolf^c^, Ardeshir Hakami-Kermani^c^, Daniel Nicastri^c^, Dong-Seok Daniel Lee^c^, Kimberly J Song^c^, Brian Housman^c^, David F Yankelevitz^a^, Emanuela Taioli^c^, Claudia I Henschke^a^, Raja M Flores^c^ for the IELCART Investigators

*Both first authors

**^a^**Department of Diagnostic, Molecular, and Interventional Radiology, Icahn School of Medicine at Mount Sinai, New York, NY, 10029, USA

**^b^**Department of Oncology, Centre Hospitalier Universitaire Vaudois (CHUV); Lausanne University, Lausanne, Switzerland

**^c^**Department of Thoracic Surgery, Icahn School of Medicine at Mount Sinai, New York, NY, 10029, USA

^d^Both first authors.

IELCART Investigators

| Raja M | Flores |
| --- | --- |
| Andrew J | Kaufman |
| Dong-Seok | Lee |
| Daniel | Nicastri |
| Andrea | Wolf |
| Kimberly | Song |
| Kenneth | Rosenzweig |
| Robert | Samstein |
| Pinaki | Dutta |
| Jorge | Gomez |
| Mary Beth | Beasley |
| Maureen | Zakowski |
| Michael | Chung |
| David F | Yankelevitz |
| Claudia I | Henschke |
| Emanuela | Taioli |
| Yeqing | Zhu |
| Natela | Paksashvili |
| Lijing | Zhang |
| Lyu | Lyu |
| Huiwen | Chan |
| Jeffrey | Zhu |
| Sydney | Kantor |
| Lauren | Lentini |
| Ardeshir | Hakami-Kermani |
| Arzu | Buyuk |
| Adie | Friedman |
| Ronald | Dreifuss |
| Stacey | Verzosa |
| Mariya | Yakubov |
| Karina | Alferdova |
| Artit | Jirapatnakul |
| Rowena | Yip |
